# Selection of a Location for Practice

**Published:** 1909-07-15

**Authors:** N. S. Hoff


### SELECTION OF A LOCATION FOR PRACTICE.

BY N. S. HOFF, D.D.S.

Lecture delivered to Students of the University of Michigan.

### PART ONE.

Almost every student wants to locate in the best city or place he can find, and I want to give you some ideas as to the methods that may be used in determining the value of different locations.

I have already called to your attention the population of several states and countries and the number of dentists in each in proportion to their population. I want to give you some statistics that I have culled from these same sources of information as to the number of dentists in the more important cities of the country.

The two cities in this country best supplied with dentists are Birmingham, Alabama and Los Angeles, California.

I do not know why Birmingham has so many more dentists than other cities, except that it is a large manufacturing city of rapid growth and a large number of people have moved there from the East and North and with them apparently a large number of dentists. In that city they have one dentist to every 600 people, and this same proportion obtains in Los Angeles, California. In this latter city there is a situation that is peculiar, due to the fact that Los Angeles is not an ordinary city. It is made up of people of more or less means, and culture who have gone there from all parts of the United States to secure health and engage in some light business occupations, consequently it is made up of a class of people that require professional services. In this part of the country there is a much larger proportion of medical men than in almost any other part of the United States, and this probably accounts for the fact that they have a larger proportion of dentists also. The same explanation will hold good relatively for such places as Washington, D. C., Denver, Colorodo, San Francisco, California, and Boston, Mass.

While these places are not known as health resorts, Boston and Washington being places of culture and of means. Another fact that should be taken into consideration is that both of these cities have a number of dental colleges, and some graduates of the schools naturally locate there. Denver and San Francisco are both attractive residence cities. One is a health resort and the other a city progressive in business and populated with a more cultured class of people than the average city, which may account for one reason why these cities support a larger number of dentists than most others. San Francisco and Denver each have one dentist to every 900 people.

Nashville, N. C., and Atlanta, Ga., each have one dentist to every 1000 people. The only explanation I can give is that while in both of these cities there is a large population of colored people, they are cultured and prosperous as there are a large number of higher educational institutions for both races in these cities. Atlanta, Ga., is one of the best business cities of the South, and many people of refinement live in that city.

There is an average of one dentist to every 1300 people in Richmond, Va., Philadelphia, New Haven and Chicago. It is a little difficult for one to reason out why these cities should be classed so near together. Richmond is an old city. There are a large number of colored people in Richmond, and why it should correspond with a city like Philadelphia is one thing that I cannot quite understand.

Philadelphia is a city which contains a large number of foreigners and transient residents. It is a large manufacturing center and a large number of foreigners are employed in its factories and as unskilled laborers.

New Haven is the city where Yale college is located and is known as a great educational center, at the same time it is the metropolis of the State of Connecticut, which is one of the most prominent manufacturing states in the country and there are a large number of manufacturing industries in New Haven.

Why Chicago should be in the same class with these cities probably will be a little difficult to determine. It is a city of large foreign population, and the people have not been educated to care for their teeth, but at the same time it is one of the most enterprising cities in the country. It is largely composed of people who think a great deal of their health and do a great deal to take care of themselves in order that they may engage in business. It is also an educational center. We have three important dental colleges located in that city and the influence of these schools on its people has probably done more to raise the standard of professional services there than any other one factor.

The next classification of one dentist to every 1500 people includes most of the cities in the country and seems to be the standard of general average. Detroit, Kansas City, Cleveland, Cincinnati, Omaha and Pittsburgh, and cities of that class fall into this class. In Minneapolis we have one dentist to every 1800 people. Just why this city should not be classified with Detroit and Cleveland I cannot quite understand. While Minneapolis has not as large a foreign population as Cleveland and Cincinnati it has a population of more sturdy Europeans who possibly do not need as much dental service as some other classes of people. It is as cultured as any of the other cities, it must be due largely to the fact that the kind of people who have gone into that country, do not require much dental service. The contributing country surrounding every place may have its influence.

In New Orleans, we find that the proportion is increased and there is one dentist to every 2,000 people. There are foreigners from all countries of the world in New Orleans, and many do not remain long enough in the city to become permanent residents.

In New York we have a still larger increase. There is one dentist to every 2,300 people. There would seem to be more room for dentists than in almost any other city in the country. There seem to be enough dentists there, however, to care for all the practice required. There is probably no city in the country in which there is more rank advertising than in New York. The city is filled with all sorts of advertisements. You will find in every street car, elevated road, subway or much frequented place the signs of the advertising dentists. You will find on the streets men in livery standing at the doors soliciting business from the sidewalk. Chicago and New York are the only cities in which I have encountered this form of advertising in such gross form.

In Jersey City there is apparently only one dentist to every 4,200 people, but in fact there is not this dis-proportion. In Jersey City a large number of its people go to New York for such professional service. They have also a large population of foreigners who are wholly ignorant of the value of dental service, they have been dumped from steamship lines and do not become permanent residents.

These statistics, perhaps, will give you a basis of one kind on which we may determine where there is a suitable opening, but you cannot pick out a location entirely on this basis. It may be valuable, if you are thinking of locating in a particular town, to find out how many dentists are practicing in that town. It may give you a clue, but it is not an infallible guide. You cannot judge of a location by these figures alone.

I shall try to classify the different fields for location into three classes. I have done so that I may give you my ideas in regard to what places are likely to need further dental service.

PART TWO.

There are three classes of places, the village, town and city. I want to consider the village as it is the most neglected opening at this time.

I would classify every collection of residents of, say, from 300 to 1,200, as a village. I have thought that many times I would like to go into the country, have a little garden and live a quiet, easy life, instead of the somewhat strenuous one I find here. I believe I could thoroughly enjoy that kind of an existence for a time at least. I have seen them in New England, that it seems to me would be real havens of rest, and I never see one of these communities but I find myself envying their village doctor, blacksmith, shoemaker, postmaster, etc. and I wishing I could settle in one of these communities and live as they do. They probably do not have as much excitement as we get in the city, in the way of football games, and general rioting, but they enjoy themselves. One of the finest men I ever knew practiced all his life in a small college town. There were not over 1,000 residents in the town, but there was a small college there, and he did all the dental work for the people of the town and the country for miles away and was wel known throughout the State and was honored and respected everywhere. But he chose to live in a small place when he might just as well have lived in a city like Chicago.

It is possible to locate in a village and to come into a lucrative practice sooner than in a city. The fortunes are not always made in cities, although it would seem the opportunities are greater. The two most celebrated surgeons of to-day, who are making greater success as practitioners than any others in the world are the Mayo Bros. of the town of Rochester, Minn.; they are reported to be making $500,000 a year. They get patients from all over the world. They are, it is true, geniuses, and exceptions. You must not expect however, should you locate in a small place, that you are going to duplicate their success in the practice of dentistry. You can locate, however, in a village of the right sort and live comfortably and easily and have time to cultivate your profession. You will find more time for doing the things that will bring you the most satisfaction.

In a place of this sort, you may have to pay as much as $5.00 per month office rent. I know a dentist in New York city who pays $4,000.00 per year for office rent. He has got to earn fifteen dollars every working day for his landlord, and every time he is sick or away from his office he is out a large amount of money. Then the other necessary expenses will be nearly as much, he receives, however large fees for his services. It may well be questioned whether after all the village man does not get better compensation than the man in the city who has to work so much harder to pay his rent and running expenses. Think what the city dentist has to earn before he can have anything for himself. Of course the city dentist will have assistants, but he has all the responsibilities and worry of the whole practice. It is a great nervous strain on a man to carry on a business of that sort. One can live a lot easier and longer in a village than in a city and not have to make so much money, but in the end he will be better off.

I know of one of our graduates who after he had been out in the world for several years came here and studied dentistry. During holidays and vacations he earned money in any way that he could. He came here with $1,500 and when he went away he had almost as much money as he brought with him. While in school he did all sorts of things to earn money. He was one of the best students we had. After graduating he went to visit his parents who lived in a small town. The following day one of the neighbors required some dental service. He fixed up a rocking chair for an operating chair and began work and has never left that place from that day to this. At that time there were not over 500 people in the town and there are not, I suppose, over 600 people there now. He started to work in his father's parlor. To-day he owns a large brick block, is a stockholder in the bank and is one of the big men in the town, honored and respected by all. He has a great reputation as a dentist. He seldom takes a vacation except to attend a dental society, and he has all the business he can attend to. He gets good fees for all his work in that little town, as good as they do in Grand Rapids and many other cities near by. He does his work conscientiously and well and people all around there are willing to come to him for his services and to pay him for them. He gets a dollar for extracting a tooth, where he gives an anesthetic, where an ordinary dentist who used to come to that town charged 25 cents. It is not always the place that gives the opportunity; the man must of course choose his place with discretion, but it is up to him to get all out of it that is possible. A good farmer will make money on a piece of land that many a farmer would starve on. A village of from 400 to 500 people will furnish work enough to keep any one busy for two or three years, and such places furnish a good opportunity for a young man with limited means to get a good start. You can get a lot of experience in that time in a village practice. You will also find conditions for treatment that you will not likely meet in a city limited practice and you will many times get a better experience in a village of that sort than you can in a city. If you get a difficult case in a city you can and probably would send it to some one else, or you will call in a dentist next door to give you advice. If you are located in a village you are thrown upon your own resources, you must develop character as well as skill.

I do not wish any of you to think I am advising you to isolate yourselves that you may rust out and stop your development, by a life of idleness or dissipation, but if you should locate in such a place, make up your mind that you have an opportunity of adding to your education and of placing yourselves in a position of power and influence in that community. Do not despise these seeming small opportunities. I believe they offer most promising fields of usefulness for the young man who can spend three or four years in a place of that sort and do so with great profit to himself.

Everything depends upon the way in which you develop the field or the way in which you occupy it as to the measure of your success or failure. The possibilities of doing good and occupying it to the best advantage are almost unlimited, and it should not be considered too much in the light of the quality or number of people you have to serve. It is possible to make a disastrous failure in such a place. You can easily become a loafer in such a place.

I would not advise you to locate in a village where the people are destitute, not only as to means of living, but destitute as to ordinary intelligence and lacking in appreciation of the best things in life. You will find some village of quick growth from some speculative incident, such towns are not likely to be of any real worth. You will find in these towns a class of people that will not want much of any thing done in the way of dentistry, and what they do have done they will not care to pay for. They are a careless sort of people who live a reckless life, who eat and drink up all they earn, and such a community is not a desirable one in which to live. There are no schools or churches, etc., and the people are not refined nor agreeable. If a town of that sort had 3,000 population, I would much rather choose a town with no more than 500 people, where there were social advantages, and where the people were of average culture and intelligence. You will find that you will do much better for yourself intellectually and morally in an intelligent small village than in a larger boom town where you might possibly make more money.

To the student, who asks me where to locate, I always ask him first where he wants to live. I should not want to live in a community where the town is made up of saloons, beer gardens, and nickle theatres. I would go to a community where the people respect themselves. I would not care to live where I would not want my family to live, and where I could not cordially associate with the people.

A community made up largely of retired farmers is not the most desirable place to locate, as they are as a rule close fisted chaps and do not want to pay much for dental services. A small town where everybody does something for a living is much better, the more industries the better. I would select a village of that sort much sooner than I would a dead one or one which had a mushroom growth and been boomed for five or six years and had begun to retrograde. Your choice ought to be governed somewhat by your own inclinations. Such a place as might strike your fancy I would not choose, nor perhaps be inclined to advise you to choose. If you choose a decadent town you will not collect one-half your bills as the people will be coming and going, and you will have no permanent practice, and the people you have to do business with are not people you would want to invite into your home. You might go there and stay for sixty to ninety days and clean up all the money you can and then go to some other town, but do not go to such a town with the idea of doing any good to the community or getting any good out of it yourself. The same thing applies to cities and larger towns. You will have that same thing to decide in the larger places.

The village then if well peopled and supported by industrious and intelligent inhabitants, promises a reasonable support; opportunity to live a simple and happy life; the possibility of self-culture; and opportunity for useful service.

